# Interfraction positional variation in pancreatic tumors using daily breath‐hold cone‐beam computed tomography with visual feedback

**DOI:** 10.1120/jacmp.v16i2.5123

**Published:** 2015-03-08

**Authors:** Mitsuhiro Nakamura, Mami Akimoto, Tomohiro Ono, Akira Nakamura, Takahiro Kishi, Shinsuke Yano, Manabu Nakata, Satoshi Itasaka, Takashi Mizowaki, Keiko Shibuya, Masahiro Hiraoka

**Affiliations:** ^1^ Department of Radiation Oncology and Image‐applied Therapy Graduate School of Medicine, Kyoto University Kyoto Japan; ^2^ Division of Clinical Radiology Service Kyoto University Hospital Kyoto Japan; ^3^ Department of Radiology Kurashiki Central Hospital Kurashiki Japan; ^4^ Department of Radiation Oncology Graduate School of Medicine, Yamaguchi University Yamaguchi Japan

**Keywords:** pancreatic cancer, breath‐hold cone‐beam computed tomography, visual feedback, interfraction positional variation

## Abstract

We assessed interfraction positional variation in pancreatic tumors using daily breath‐hold cone‐beam computed tomography at end‐exhalation (EE) with visual feedback (BH‐CBCT). Eleven consecutive patients with pancreatic cancer who underwent BH intensity‐modulated radiation therapy with visual feedback were enrolled. All participating patients stopped oral intake, with the exception of drugs and water, for >3 hr before treatment planning and daily treatment. Each patient was fixed in the supine position on an individualized vacuum pillow. An isotropic margin of 5 mm was added to the clinical target volume to create the planning target volume (PTV). The prescription dose was 42 to 51 Gy in 15 fractions. After correcting initial setup errors based on bony anatomy, the first BH‐CBCT scans were performed before beam delivery in every fraction. BH‐CBCT acquisition was obtained in three or four times breath holds by interrupting the acquisition two or three times, depending on the patient's BH ability. The image acquisition time for a 360° gantry rotation was approximately 90 s, including the interruption time due to BH. The initial setup errors were corrected based on bony structure, and the residual errors in the target position were then recorded. The magnitude of the interruptions variation in target position was assessed for 165 fractions. The systematic and random errors were 1.2 and 1.8 mm, 1.1 and 1.8 mm, and 1.7 and 2.9 mm in the left–right (LR), anterior–posterior (AP), and superior–inferior (SI) directions, respectively. Absolute interfraction variations of >5 mm were observed in 18 fractions (11.0%) from seven patients because of EE‐BH failure. In conclusion, target matching is required to correct interfraction variation even with visual feedback, especially to ensure safe delivery of escalated doses to patients with pancreatic cancer.

PACS number: 87.57.Q‐, 87.57.‐s, 87.55.Qr

## I. INTRODUCTION

The National Cancer Institute reported that an estimated 46,420 new cases and 39,590 deaths from pancreatic cancer occurred in 2014 in the United States.^(1)^ Surgical resection is the mainstay of curative treatment associated with long‐term survival in patients with pancreatic cancer; however, most patients present with locally advanced, unresectable, or metastatic disease at diagnosis.^(2)^ Although radiation therapy has been an important option for these patients, radiation therapy for pancreatic cancer is highly toxic in some cases, partly because of the high dose to the surrounding organs at risk (OARs).^3^, ^4^ Severe gastrointestinal (GI) toxicity is generally related to high‐dose volumes in the stomach and bowels.^3^, ^4^

One method by which to reduce the high dose to such organs is management of respiratory motion.^5^, ^6^ Several researchers have reported that pancreatic tumor motion due to breathing exceeded 10 mm using cine magnetic resonance images, four‐dimensional computed tomography (CT), electromagnetic transponders, and cone‐beam CT (CBCT).^7^, ^8^, ^9^, ^10^, ^11^, ^12^, ^13^, ^14^ Without respiratory management, a large planning target volume (PTV) is needed to cover such internal motion, resulting in inclusion of a large volume of OARs.

To achieve dose escalation in patients with locally advanced unresectable pancreatic cancer, we applied hypofractionated intensity‐modulated radiotherapy (IMRT) combined with breath hold (BH) at end‐exhalation (EE) using visual feedback. Our previous study concluded that a margin size of 5 mm was needed to cover the 95th percentiles of the overall positional variations, including the intra‐ and interfraction positional variations, under EE‐BH conditions with visual feedback in ten patients.^15^, ^16^ However, the intra‐ and interfraction positional variations were based on three repeat CT scans at an interval of one to two weeks during the chemoradiation course. GI content, bowel gas, and other GI states generally vary with time, potentially affecting pancreatic tumor positions.^17^, ^18^, ^19^, ^20^, ^21^ Therefore, it is important to verify the daily target position, especially in hypofractionated radiotherapy.

The recent introduction of soft‐tissue imaging to the treatment room offers the possibility of daily imaging and online correction of target position errors before treatment. CBCT imaging is suitable for online correction of tumor position errors. Whitfield et al.,^(11)^ van der Horst et al.,^(13)^ and Solla et al.^(14)^ reported the use of CBCT to assess interfractional position changes of pancreatic tumors under free breathing. However, there have been no reports of interfraction positional variations in pancreatic tumors using BH‐CBCT at EE.

The purpose of this study was to assess the interfraction positional variations in pancreatic tumors using daily BH‐CBCT at EE with visual feedback during BH‐IMRT.

## II. MATERIALS AND METHODS

### A. Patients

Eleven consecutive patients who underwent BH‐IMRT for locally advanced unresectable pancreatic cancer between January 2012 and November 2014 were enrolled in an institutional review board‐approved trial. These patients met the following eligibility criteria: 1) performance status of 0 or 1; 2) pancreatic tumor motions of ≥10 mm in the superior–inferior (SI) direction under free breathing as observed with four‐dimensional CT; 3) ability to hold breath at EE for approximately 20 s; and 4) the completion of written informed consent. All participating patients stopped oral intake with the exception of drugs and water for ≥3 hr before treatment planning and daily treatment. No patient was provided oxygen during the BH‐CT and BH‐CBCT scan. Patient characteristics are shown in Table 1. A brochure on BH‐CT scanning using visual feedback was distributed to all participating patients by the day of CT simulation. If necessary, they practiced BH with medical staffs.

**Table 1:** Patient characteristics

*Patient*	*Sex*	*Age (yr)*	*TNM*	*Location*	*Fiducial Marker*	*Dose (Gy)*
1	M	76	T3N0M0	Body of pancreas	Visicoil	45
2	F	69	T4N0M0	Head of pancreas	None	48
3	M	43	T4N0M0	Head of pancreas	None	48
4	M	78	T4N0M0	Head of pancreas	None	48
5	F	69	T4N0M0	Head of pancreas	None	51
6	F	57	T3N0M0	Head of pancreas	None	51
7	M	54	T3N0M0	Head of pancreas	None	42
8	F	65	T4N0M0	Body of pancreas	None	51
9	M	63	T4N0M0	Head of pancreas	None	42
10	F	53	T4N1M0	Body of pancreas	None	42
11	M	72	T3N0M0	Body of pancreas	None	42

M=male; F=female.

### B. Breath‐hold planning CT scans

During planning simulation, all participating patients were positioned and immobilized on an individualized vacuum pillow (BodyFIX; Medical Intelligence, Schwabmünchen, Germany) with both arms raised. A marker block with two infrared reflecting dots was placed tightly on the anterior abdominal surface of the patient. A Real‐Time Position Management (RPM) system (Varian Medical Systems, Palo Alto, CA) illuminated and monitored anterior–posterior (AP) abdominal skin surface displacement. The abdominal motion signal gave visual feedback to the patient with the aid of video goggles.^(15)^ The patients were asked to breathe following simple audio instructions, such as “breathe in, breathe out, and hold your breath”, while watching their abdominal displacement with the goggles. During BH, they held their breath at EE for approximately 20 s, depending on their BH ability. The scan protocol for planning simulation was as follows. First, two scout views were taken under EE‐BH conditions to determine the scan start position. Second, the whole abdomen, from the superior border of the liver to the iliac crest, was scanned in helical mode with a 16 slice CT scanner (LightSpeed 16RT; General Electric Medical Systems, Waukesha, WI) under EE‐BH conditions with visual feedback, which took approximately 10 s. The acquisition parameters of the helical CT scan were a rotational time of 0.7 s and a helical pitch of 27.5 mm/rotation. BH‐CT data were reconstructed in a field of view (FOV) of 550 mm with a slice thickness of 2.5 mm. Finally, contrast‐enhanced BH‐CT data were acquired for the same scanning length in helical mode. Iodinated contrast medium (300 mg/ml) was infused at a rate of 2 ml/s. The patients received a total contrast medium volume based on twice their body weight (up to 100 ml). The delay time between the injection of the contrast medium and the start of the contrast‐enhanced BH‐CT scan was set at 40 s. The technical details have been previously described.^(15)^

### C. Target delineation

The gross tumor volume (GTV), defined to be the envelope of the GTV in each BH‐CT dataset, was delineated manually. The GTV included reproducibility errors of two BH‐CT scans. The clinical target volume (CTV) was defined as the GTV plus a 5 mm isotropic margin. The CTV also included the retropancreatic space between the root of the celiac trunk and the superior mesenteric artery. The PTV was determined by adding a 5 mm isotropic margin to the CTV based on the result of our previous study.^(15)^ All target delineations were performed by one radiation oncologist (A.N.) and reviewed by one expert (S. I.).

### D. Setup error corrections and image registration

Patients were first aligned based on tattoos on the skin indicating the planning isocenter location with a system of wall‐mounted alignment lasers. A pair of orthogonal kV X‐ray planar images was then obtained using the on‐board imager systems of a Clinac iX Linear Accelerator (Varian Medical Systems). These kV X‐ray planar images were semiautomatically aligned to their corresponding digitally reconstructed radiographs. After correcting initial setup errors based on bony anatomy, the first BH‐CBCT scan was acquired in half‐fan mode with a bow‐tie filter. BH‐CBCT acquisition was obtained in three or four breath holds, each approximately 15 to 20 s, by interrupting the acquisition two or three times depending on the patient's BH ability. The image acquisition time for a 360° gantry rotation was approximately 90 s including the interruption time of BH. The BH‐CBCT data were reconstructed in a FOV of 450 mm with a slice thickness of 2.5 mm.

Image registrations were performed using the 3D fusion feature in Varian Offline Review (ver. 11). The residual errors in target position were then recorded using the first BH‐CBCT images. The residual errors were defined as the distance GTV centroid position in 3D derived from the first BH‐CBCT images relative to the corresponding BH‐CT images. Target matching was performed based on the GTV and/or the surrounding structures such as the stomach, duodenum, major vessels, or areas of flat. Radiation oncologists and a medical physicist specializing in pancreatic cancer established the target matching protocol. A single trained observer conducted the target matching, and the matching results for all patients were assessed by other two medical physicists who were present during the daily treatments.

### E. Interfraction positional variations

The means and standard deviations (SDs) of the pancreatic tumor positional errors were calculated for each patient in the left–right (LR), AP, and SI directions. From these values, the population systematic error (Σ) and random error (σ) were calculated for each direction. The Σ was calculated as the SD of the mean displacement for each individual patient. The σ was determined by computing the root mean square of the SD of an individual patient's displacements. A total of 165 fractions (11 patients×15 fractions) were analyzed.

## III. RESULTS

**Figure 1:**
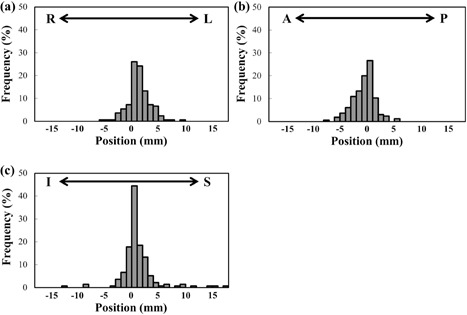
Frequency distributions of the interfraction positional variations in pancreatic tumors using daily breath‐hold cone‐beam computed tomography with visual feedback. The mean±SD of interfraction positional variation was 0.9±2.1 mm (range, ‐6.0 to 9.0 mm), −1.1±2.1 mm (range, ‐8.0 to 5.0 mm), and 0.6±3.3 mm (range, ‐13.0 to 17.0 mm) for all fractions in the (a) left–right, (b) anterior–posterior, and (c) superior–inferior directions, respectively. Positive values indicate the left, posterior, and superior directions.

The maximum interobserver variation in all cases was within 2 mm. Figure 1 shows the frequency distribution of the interfraction positional variations. The mean±SD of the interfraction positional variation for all fractions was 0.9±2.1 mm (range, ‐6.0 to 9.0 mm), −1.1±2.1 mm (range, ‐8.0 to 5.0 mm), and 0.6±3.3 mm (range, ‐13.0 to 17.0 mm) in the LR, AP, and SI directions, respectively. Positive values indicate the left, posterior, and superior directions.

**Figure 2:**
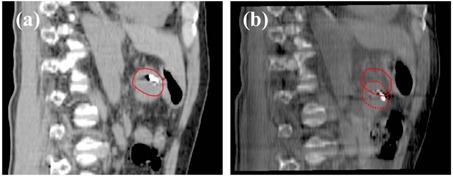
An example of target displacement in the sagittal plane on (a) planning breath‐hold computed tomography, and (b) breath‐hold cone‐beam computed tomography. The solid and broken lines show the outline of the gross tumor volume at (a) simulation and at (b) treatment, respectively. Note that the gross tumor volume on the breath‐hold cone‐beam computed tomography image was deviated from the planning gross tumor volume because of breath holding at end‐exhalation failure.

Table 2 shows the means±SDs of the interfraction positional variations for each patient. The mean values were within 5 mm, except for one patient (Patient 10). Table 3 summarizes the deviation rate of the interfraction positional variations in each direction for each patient. Absolute interfraction variations of >5 mm were observed in 18 fractions (11.0%) from seven patients. Of those, the interfraction variations of ≥10 mm were observed in 5 fractions (3.0%) in the SI direction from two patients. Figure 2 shows an example of target displacement in the sagittal plane. Note that the GTV on the BH‐CBCT image was deviated from the planning GTV because of EE‐BH failure. The values of Σ and σ were 1.2 and 1.8 mm, 1.1 and 1.8 mm, and 1.7 and 2.9 mm in the LR, AP, and SI directions, respectively.

**Table 2:** Means±SDs of the interfraction positional variations in pancreatic tumors using daily breath‐hold cone‐beam computed tomography with visual feedback for each patient. Positive values indicate the left, posterior, and superior directions

*Patient*	*LR (mm)*		*AP (mm)*		*SI (mm)*	
	*Mean*	*SD*	*Mean*	*SD*	*Mean*	*SD*
1	−0.2	3.0	−1.0	1.4	0.0	5.8
2	2.5	1.7	−0.6	1.9	−0.2	1.7
3	3.3	2.3	−1.3	2.1	0.5	1.6
4	−0.5	1.9	−2.0	1.9	0.4	1.1
5	1.5	1.2	0.2	1.6	0.1	0.8
6	1.1	1.9	−3.3	1.7	0.3	1.3
7	−0.2	0.9	−1.5	2.1	0.4	2.4
8	0.1	1.5	−0.5	0.9	1.4	2.3
9	0.8	1.1	0.6	0.9	−0.7	1.0
10	0.6	2.5	−2.1	3.1	5.5	5.5
11	0.3	0.6	−0.3	1.3	−0.9	2.9

SD=standard deviation; LR=left–right; AP=anterior–posterior; SI=superior–inferior.

**Table 3:** Deviation rate of the interfraction positional variations in pancreatic tumors using daily breath‐hold cone‐beam computed tomography with visual feedback for each patient. The unit of numbers listed is in fraction number and the percentage rate listed in the parentheses is compared to 15 fractions

	*LR*		*AP*		*SI*	
	>5 mm,		>5 mm,		>5 mm,	
*Patient*	<10 mm	≥10 mm	<10 mm	≥10 mm	<10 mm	≥10 mm
1	1 (6.7%)	0 (0.0%)	0 (0.0%)	0 (0.0%)	1 (6.7%)	2 (13.3%)
2	0 (0.0%)	0 (0.0%)	0 (0.0%)	0 (0.0%)	0 (0.0%)	0 (0.0%)
3	2 (13.3%)	0 (0.0%)	0 (0.0%)	0 (0.0%)	0 (0.0%)	0 (0.0%)
4	0 (0.0%)	0 (0.0%)	0 (0.0%)	0 (0.0%)	0 (0.0%)	0 (0.0%)
5	0 (0.0%)	0 (0.0%)	0 (0.0%)	0 (0.0%)	0 (0.0%)	0 (0.0%)
6	0 (0.0%)	0 (0.0%)	2 (13.3%)	0 (0.0%)	0 (0.0%)	0 (0.0%)
7	0 (0.0%)	0 (0.0%)	0 (0.0%)	0 (0.0%)	1 (6.7%)	0 (0.0%)
8	0 (0.0%)	0 (0.0%)	0 (0.0%)	0 (0.0%)	1 (6.7%)	0 (0.0%)
9	0 (0.0%)	0 (0.0%)	0 (0.0%)	0 (0.0%)	0 (0.0%)	0 (0.0%)
10	1 (6.7%)	0 (0.0%)	2 (13.3%)	0 (0.0%)	3 (20.0%)	3 (20.0%)
11	0 (0.0%)	0 (0.0%)	0 (0.0%)	0 (0.0%)	1 (6.7%)	0 (0.0%)

LR=left–right; AP=anterior–posterior; SI=superior–inferior.

## IV. DISCUSSION

IMRT techniques allow for the safe delivery of high‐dose radiation to the target while sparing adjacent OARs. Dose gradients become steeper for hypofractionated IMRT than for conventional fractionated IMRT. Because the pancreatic cancer is surrounded by radiosensitive OARs, mistarget localization causes the unintentional delivery of high doses to the OARs. In the present study, we focused on the interfraction positional variations in the pancreatic tumors using daily BH‐CBCT with visual feedback during BH‐IMRT. Our results confirmed the high reproducibility of the pancreatic tumor position using visual feedback.

To allow for target matching on CBCT images, CBCT image quality is crucial. Duggan et al.^(22)^ stated that BH‐CBCT for patient setup was feasible in lung stereotactic body RT. Using the same technique as that used by Duggan et al., we divided one gantry rotation into three to four segments to acquire BH‐CBCT images, depending on the patient's BH ability, because BH at EE for one rotation time of 60 s was impossible for most patients. As shown in Fig. 2, the CBCT image quality acquired under BH conditions was relatively sharp in the present study.

Even when dividing one rotation into several segments, a reduction in image quality due to low reproducibility of the target cannot be observed. Our previous study showed that visual feedback provided high intra‐BH reproducibility of the pancreatic tumor.^(15)^ Thus, some artifacts caused by low pancreatic tumor position reproducibility were not observed when matching the target on BH‐CBCT images. Compared with interobserver variations using implanted markers,^(13)^ our interobserver variations based on anatomical structures were slightly larger, but clinically within an acceptable range.

**Figure 3:**
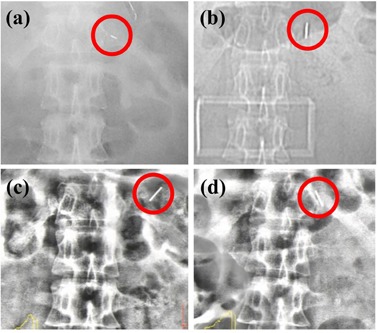
Interfractional migration and rotation of a Visicoil implanted around the pancreatic tumor: (a) at implantation, (b) 1 week after, (c) 3 weeks after, and (d) 5 weeks after. Visicoil is shown in the circle. The marker frequently migrated and rotated.

In the present study, we compared the target on BH‐CBCT images with that on planning BH‐CT images based on anatomical structure, even for the patient with the implanted marker. Markers implanted either in the tumor itself or nearby are often used as the internal surrogate with which to localize the tumor position.^13^, ^14^ Although van der Horst et al.^(13)^ stated that small changes in the pair distance of implanted markers over the course of treatment may be attributed to tissue deformation rather than to marker migration, we have previously experienced large marker migration. (This case was not a participating patient in the present study.) Figure 3 shows that the implanted fiducial marker around the pancreatic tumor frequently migrated and rotated, which may have been caused by flat layer deformation or changes in GI states. Note that such implanted markers are of no use with target matching; therefore, target matching is required to correct interfraction variation, even with implanted markers.

Several investigators have examined interfraction positional variation of the pancreas using a variety of methods;^12^, ^13^, ^14^, ^19^ however, most examinations were performed under free breathing. van der Horst et al.^(13)^ demonstrated large interfractional pancreatic position variation of >10 mm using fiducial markers visible on daily CBCT scans. Shinohara et al.^(12)^ showed that the mean absolute shifts were 4.5, 3.9, and 6.4 mm in the LR, AP, and SI directions, respectively. Meanwhile, Wysocka et al.^(20)^ employed voluntary expiration BH and showed that the 90th percentiles of the interfraction positional variations in the pancreas were 8.9, 7.9, and 23.0 mm in the LR, AP, and SI directions, respectively. Compared with the results of these studies, our method using visual feedback appeared to be effective in reducing interfraction positional variation, even using external surrogates. Stock et al.^(23)^ and Peng et al.^(24)^ showed that the introduction of video feedback improved the reproducibility of lung tumor position. Although they evaluated a different disease site, their results support ours.

Table 3 shows that the positional uncertainty in the LR and AP directions was identical, and extreme deviations (e.g., ≥10 mm) were not observed. Meanwhile, interfraction positional variations of ≥10 mm were observed in 5 fractions (3.0%) in the SI direction, although visual feedback resulted in a lower positional uncertainty in the SI direction compared with pancreatic tumor motion under free breathing. Possible causes of interfraction positional variations in pancreatic tumors involve EE‐BH failure and daily variations in the surrounding organ fillings, including the stomach and bowel.^17^, ^18^, ^19^, ^20^, ^21^ Although none of our patients consumed anything orally, with the exception of drugs or water, for ≥3 hr before each BH‐CT scan, control of such physiological phenomena would be difficult. Our group^(21)^ found that considerable interfractional movement occurred even under fasting conditions, especially in the stomach. The advantage of BH‐CBCT scans is that a possible large target displacement can be easily identified. When target displacements of >5 mm were observed on first BH‐CBCT images after bony structure alignment in clinical practice at our institution, additional BH‐CBCT scans were done to confirm the target position after the initial target matching on the first BH‐CBCT images.

Another concern is patients' cheating during BH. At out institution, in order to prevent patients' cheating, a brochure explaining BH‐CT scanning is distributed to all participating patients on the day before CT simulation. Our medical staffs provide BH for patients worrying about BH. Even with enough practice, some patients may cheat during BH. If implanted fiducial markers are used as reliable surrogates, we can check whether patients are cheating on kV or MV X‐ray images during beam delivery under BH. As another approach, several BH‐CBCT scans are also helpful in verifying the intrafraction positional variations.

Based on findings obtained from the current and our previous studies,^15^, ^16^, ^21^, ^25^ a phase I/II radiation dose‐escalation study of full‐dose gemcitabine with BH‐IMRT is ongoing in our department for patients with locally advanced unresectable pancreatic cancer, referring to the previous dose‐escalation trials of full‐dose gemcitabine with conventional RT at University of Michigan.^(26)^ Better outcomes can be expected with the use of BH‐IMRT with image guidance.

## V. CONCLUSIONS

BH‐CBCT with visual feedback generally provided high reproducibility of the pancreatic tumor position, even based on bony structure. However, absolute interfraction variations of >5 mm were occasionally observed. Therefore, target matching is required to correct interfraction variation even with visual feedback, especially to ensure safe delivery of escalated doses to patients with pancreatic cancer.
